# Vicarious Contrast Medium Excretion Mimicking Acute Hemorrhage: Don't Call the Interventional Radiologist!

**DOI:** 10.7759/cureus.115978

**Published:** 2026-09-09

**Authors:** Apostolos A Mouroukis, Maria Theodora M Moschouri, Emine E Nezntet Oglou, Kampasakal K Chousein Neslichan, Maria M Takoutsikou, Hippocrates Moschouris

**Affiliations:** 1 Diagnostic and Interventional Radiology, University Hospital of Alexandroupolis, Alexandroupoli, GRC; 2 College of Medicine, Democritus University of Thrace, Alexandroupoli, GRC; 3 Radiology and Interventional Radiology, University Hospital of Alexandroupolis, Alexandroupoli, GRC; 4 Radiology, University Hospital of Alexandroupolis, Democritus University of Thrace, Alexandroupoli, GRC

**Keywords:** coil embolization, diagnostic pitfall, digital subtraction angiography, interventional radiology, vicarious contrast medium excretion

## Abstract

Vicarious contrast medium excretion (VCME) is an uncommon phenomenon in which iodinated contrast is eliminated through non-renal pathways, predominantly via the hepatobiliary system. Although benign, it may closely mimic active gastrointestinal hemorrhage on follow-up computed tomography (CT), potentially leading to unnecessary invasive procedures.

We report the case of a 28-year-old woman with acute leukemia complicated by methotrexate-induced acute pancreatitis and massive retroperitoneal hemorrhage. Contrast-enhanced CT demonstrated findings suspicious for active arterial bleeding originating from the gastroduodenal artery territory. Subsequent digital subtraction angiography revealed no definite bleeding source, and empirical coil embolization of the gastroduodenal artery was successfully performed. Following administration of a large cumulative contrast volume and transient renal impairment requiring hemodialysis, repeat CT demonstrated hyperattenuating material within the gallbladder and multiple small-bowel loops, initially raising concern for recurrent gastrointestinal bleeding. Careful review of the unenhanced images confirmed that the hyperdense intraluminal material preceded the second contrast administration and remained unchanged on post-contrast images, establishing the diagnosis of simultaneous hepatobiliary and intestinal VCME.

This case highlights an important diagnostic pitfall following repeated contrast-enhanced imaging and endovascular intervention. Recognition of the characteristic imaging features of VCME, particularly the presence of hyperattenuating bowel contents on unenhanced CT and the absence of interval enhancement, is essential for differentiating this benign entity from true recurrent hemorrhage and for avoiding unnecessary angiography, embolization, or surgery.

## Introduction

Contrast-enhanced computed tomography (CT) plays a central role in the diagnosis and management of patients presenting with acute abdominal or retroperitoneal hemorrhage. In hemodynamically stable patients, CT angiography (CTA) accurately localizes the bleeding source and guides subsequent endovascular treatment, while digital subtraction angiography (DSA) remains the reference standard for both diagnosis and transcatheter embolization [1,2]. Empirical embolization may be performed even in the absence of angiographically visible active bleeding when CT findings, hematoma distribution, and clinical presentation strongly suggest a specific arterial source [3].

Water-soluble iodinated contrast media (CM) are almost exclusively eliminated through renal excretion under normal physiological conditions. However, under specific circumstances--including impaired renal function, transient alterations in renal perfusion, or administration of large cumulative volumes of contrast medium--alternative excretory pathways may become evident [4]. This phenomenon, known as vicarious contrast medium excretion (VCME), results from hepatobiliary secretion of iodinated contrast, with subsequent opacification of the gallbladder and, following biliary drainage, the intestinal lumen [5]. Rarely, contrast material may also accumulate within the peritoneal or pericardial spaces [6]. Although uncommon, these findings may be detected several hours after contrast administration and represent a well-described but frequently overlooked imaging phenomenon.

The radiological appearance of VCME may closely resemble active gastrointestinal hemorrhage, enteric contrast leakage, hemobilia, or other pathological processes, particularly in critically ill patients undergoing serial CT examinations. Failure to recognize this entity may result in unnecessary diagnostic, interventional, or even surgical procedures. 

Herein, we present a case of simultaneous hepatobiliary and intestinal vicarious contrast medium excretion identified on follow-up CT after empirical gastroduodenal artery (GDA) embolization for acute hemorrhage. The case highlights a potentially important diagnostic pitfall and emphasizes the imaging features that enable confident differentiation between VCME and recurrent gastrointestinal bleeding. Familiarization with these imaging features could be useful not only for radiologists but also for intensivists and surgeons, and could facilitate the appropriate management of complex cases of acute abdominal hemorrhage.

## Case presentation

A 28-year-old Caucasian woman in the intensive care unit with a recent diagnosis of acute leukemia and severe methotrexate-induced acute pancreatitis developed sudden hemodynamic instability (systolic blood pressure: 86 mmHg; heart rate: 120 beats per minute) and a decrease in hematocrit from 24.6% to 18.1%. Emergent, contrast-enhanced (120 mL of CM) computed tomography (CT) showed a massive hemorrhagic peripancreatic collection with multiple areas of active extravasation, presumably from branches of the gastroduodenal artery. Based on the CT appearance and the clinical scenario, digital subtraction angiography (DSA) was performed immediately thereafter (Figure 1).

**Figure 1:**
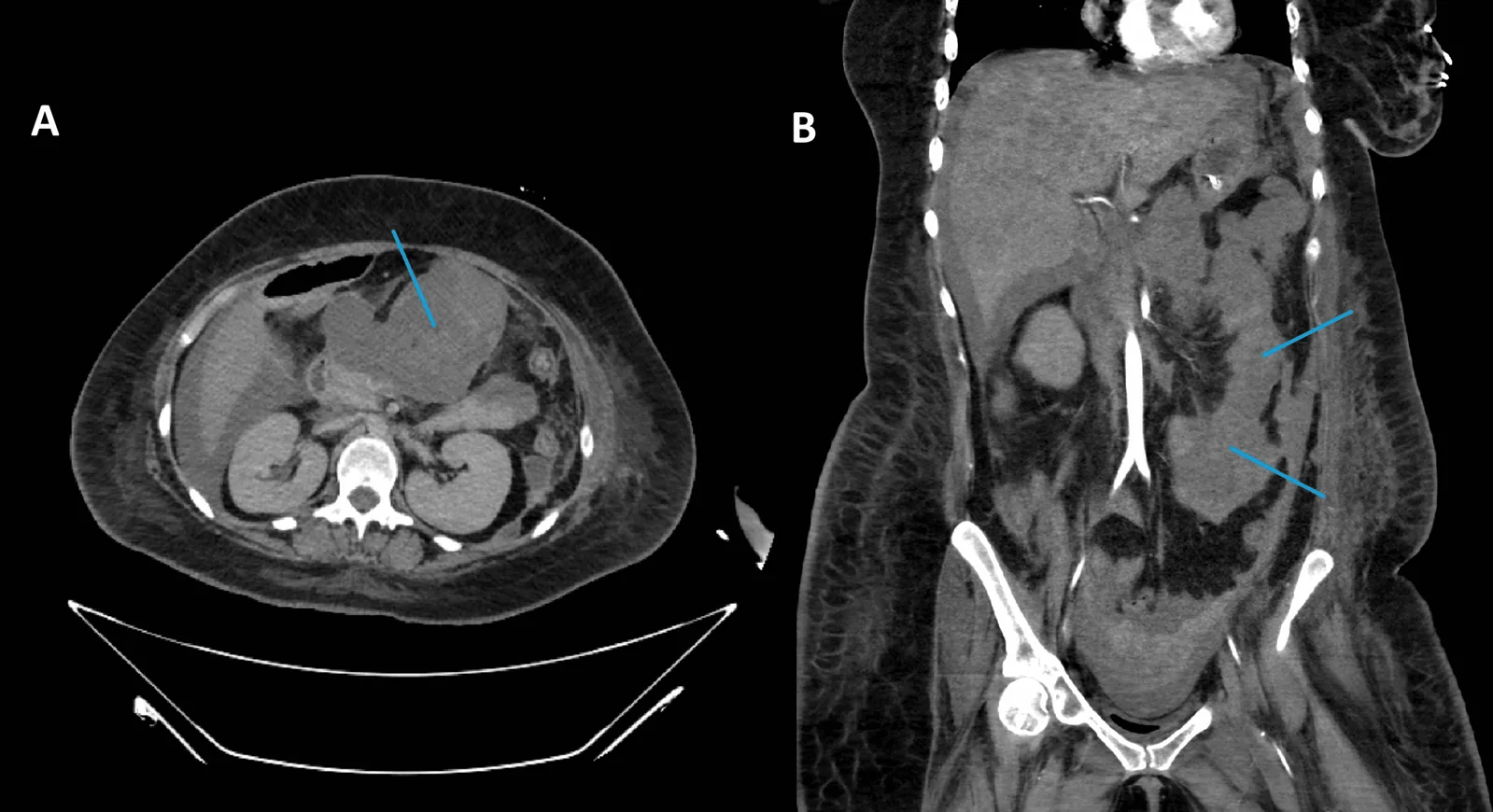
Initial contrast-enhanced CT examination demonstrating acute retroperitoneal hemorrhage. (A) Axial contrast-enhanced CT image demonstrates a large hemorrhagic peripancreatic and retroperitoneal collection with imaging findings suspicious for acute arterial bleeding. (B) Coronal reformatted CT image demonstrates the full craniocaudal extent of the retroperitoneal hematoma and its close anatomical relationship to the gastroduodenal artery territory, findings that prompted urgent digital subtraction angiography and subsequent empirical transcatheter coil embolization.

Vascular access was obtained through the right common femoral artery using a standard percutaneous approach. Selective and superselective catheterization of the celiac trunk, superior mesenteric artery (SMA), their major branches, as well as selected lumbar and intercostal arteries, was subsequently performed. Systematic angiographic evaluation failed to demonstrate active contrast extravasation, pseudoaneurysm formation, or an arteriovenous fistula despite the extensive hemorrhage demonstrated on CT.

Considering the topographic distribution of the retroperitoneal hemorrhage together with the presumed arterial source of hemorrhage, empirical transcatheter embolization of the gastroduodenal artery (GDA) was undertaken using detachable microcoils. Completion angiography confirmed successful occlusion of the embolized vessel without evidence of residual filling or immediate procedure-related complications. Preservation of the remaining splanchnic arterial circulation was confirmed.

Digital subtraction angiography was subsequently performed, followed by empirical embolization of the gastroduodenal artery based on the presumed source of hemorrhage. Selective superior mesenteric angiography demonstrates the mesenteric arterial branches without angiographically visible active contrast extravasation, pseudoaneurysm, or arteriovenous fistula (Figure 2). Superselective angiographic evaluation of the gastroduodenal/pancreaticoduodenal arterial territory again demonstrates no definite active bleeding source (Figure 3). Given the topographic distribution of the hematoma and the presumed mechanism of hemorrhage, empirical coil embolization of the gastroduodenal artery was performed. Completion angiography after coil deployment demonstrates the embolization material in the gastroduodenal arterial territory, preserved perfusion of the remaining mesenteric circulation, and no residual angiographic evidence of active hemorrhage (Figure 4).

**Figure 2:**
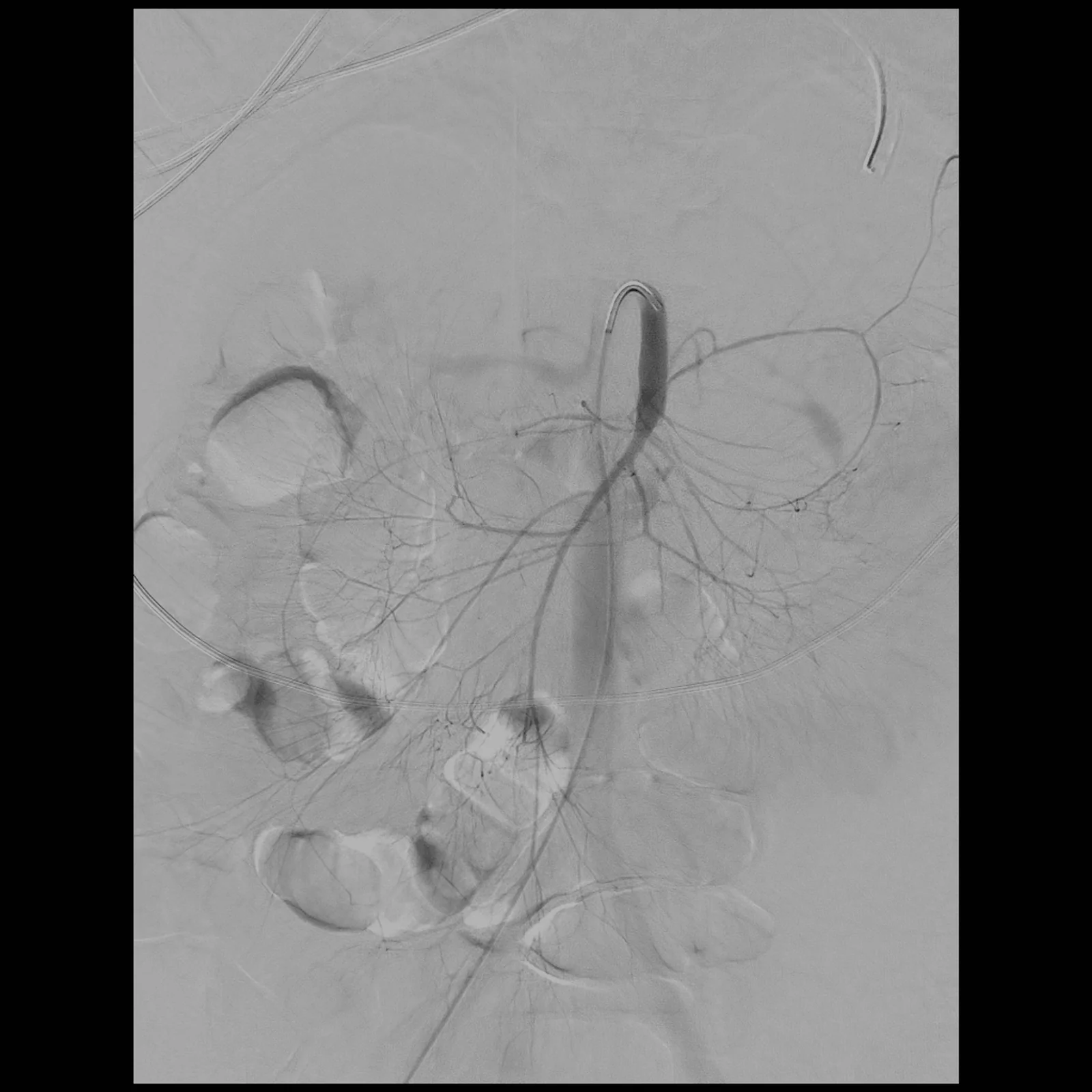
Selective superior mesenteric angiography.

**Figure 3:**
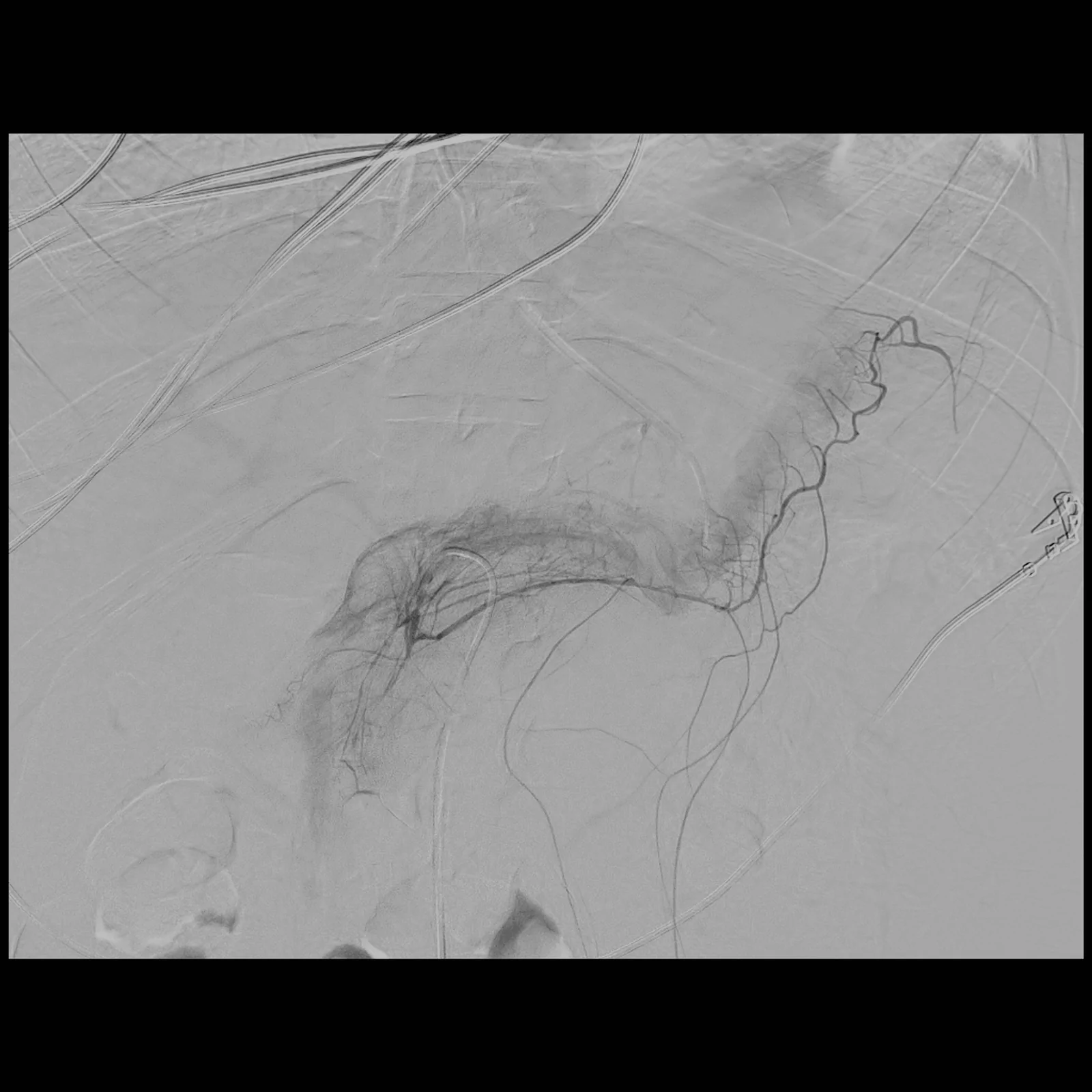
Superselective angiographic evaluation of the gastroduodenal/pancreaticoduodenal arterial territory.

**Figure 4:**
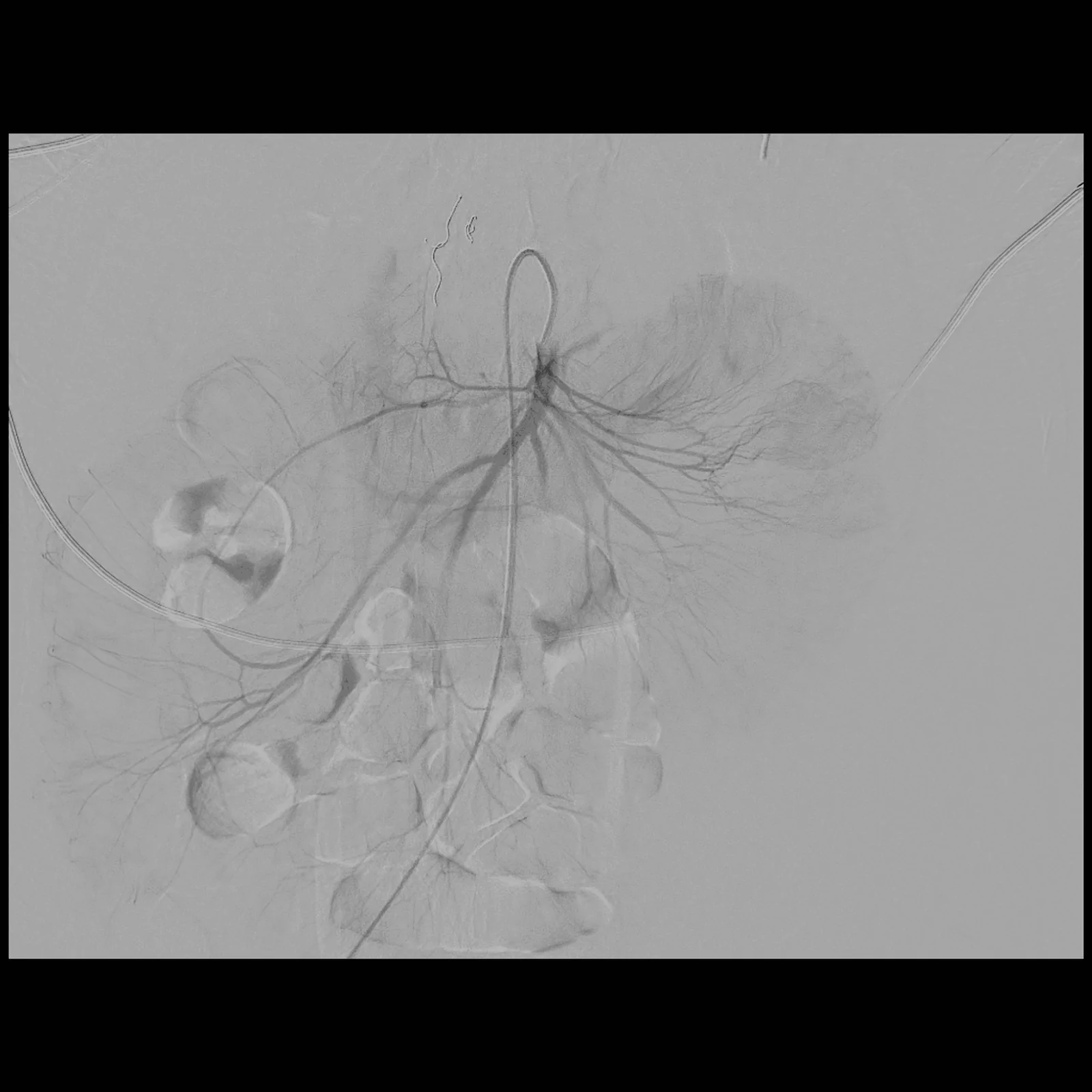
Completion angiography following coil embolization.

Hemodialysis was performed after the CT scan to compensate for renal impairment; 300 mL of intra-arterial CM was additionally required for embolization. Eight hours post-embolization, a new episode of hypotension occurred, and a second contrast-enhanced CT was performed.

The previously demonstrated retroperitoneal hematoma remained evident without convincing CT evidence of new active arterial bleeding. However, hyperattenuating material was identified within the gallbladder and in multiple small-bowel loops. At first glance, these findings raised concern for recurrent gastrointestinal hemorrhage and contrast extravasation with intraluminal filtration.

Careful review of the unenhanced CT acquisition demonstrated that the hyperdense intraluminal material was already present before intravenous contrast administration during the follow-up examination and did not increase in attenuation on the post-contrast phases (Figure 5). There was no evidence of hematemesis or melena. It was also confirmed that no oral contrast medium had been administered through the nasogastric tube.

**Figure 5:**
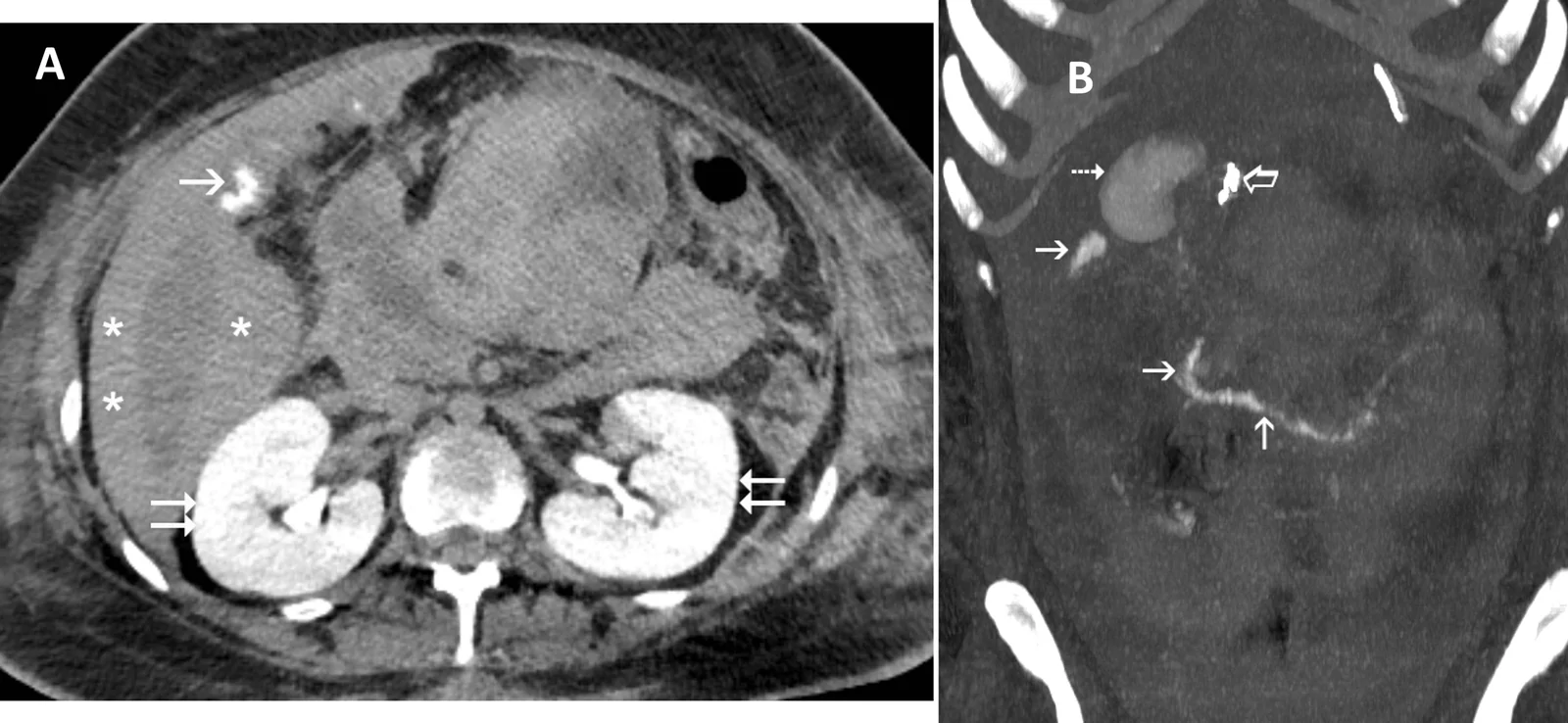
(A) Axial image from the unenhanced phase of the second CT examination demonstrates collections of contrast medium within small bowel loops (arrows), consistent with VCME, together with hyperattenuating perihepatic fluid (asterisks) and a persistent nephrogram related to renal impairment (double arrows). (B) Coronal maximum intensity projection image demonstrates small-bowel contrast (arrows), gallbladder opacification (dotted arrow), and coils in the gastroduodenal artery (open arrow). VCME: vicarious contrast medium excretion.

Correlation with the patient's clinical course and the characteristic imaging appearance supported the diagnosis of simultaneous hepatobiliary and intestinal vicarious contrast medium excretion (VCME), rather than ongoing gastrointestinal bleeding. The patient's status improved with conservative treatment in the following days.

## Discussion

Vicarious contrast medium excretion (VCME) describes the elimination of intravascularly administered iodinated contrast medium through non-renal pathways. Under normal physiological conditions, the overwhelming majority of water-soluble iodinated contrast is cleared by glomerular filtration, whereas only a very small fraction is excreted through alternative routes. Nevertheless, hepatobiliary and intestinal elimination may become conspicuous when renal clearance is delayed, the circulating contrast load is high, or renal perfusion is transiently reduced. The resulting opacification may involve the gallbladder, small bowel, colon, and, less commonly, the peritoneal or pericardial spaces [4,6].

The exact pathophysiological mechanism of VCME has not been fully established. Earlier explanations emphasized binding of contrast molecules to plasma proteins, followed by hepatocellular uptake and biliary secretion or direct enterocyte-mediated excretion [4]. Modern low-osmolar contrast agents have limited protein binding; therefore, vicarious elimination ordinarily represents less than 1% of the administered dose. However, prolonged intravascular recirculation caused by impaired renal filtration or reduced renal blood flow may increase the amount available for hepatobiliary and gastrointestinal clearance. Acute kidney injury, urinary obstruction, hemorrhagic shock, cardiac dysfunction, and a large cumulative contrast burden have all been associated with a greater likelihood of this phenomenon [7,8].

In the present case, the patient received iodinated contrast during the initial CT examination and a further intra-arterial contrast load during extensive angiographic evaluation and embolization. The examinations were performed in close temporal succession in the setting of acute hemorrhage. Even in the absence of documented advanced renal failure, this combination may prolong contrast circulation and favor non-renal elimination. Previous reports have shown that high contrast volume and hemodynamic compromise may potentiate VCME, while persistent renal enhancement may coexist as an imaging marker of delayed contrast clearance [9].

VCME is important primarily because it may imitate clinically significant pathology. Intraluminal high-attenuation material in the bowel of a patient recently treated for abdominal hemorrhage may be interpreted as active gastrointestinal bleeding, recurrent arterial extravasation, hemobilia, or a vascular-enteric fistula.

Similar appearances have led to concern for nephroenteric fistula after renal trauma and have also been reported as unexpected colonic opacification after intravenous contrast administration [10,11]. In a critically ill or recently embolized patient, such a misinterpretation may trigger repeat angiography, further embolization, endoscopy, surgery, or additional contrast exposure.

Several imaging features help distinguish VCME from ongoing hemorrhage. The most useful observation is the presence of intraluminal hyperattenuating material on the unenhanced phase of a subsequent CT examination. True active bleeding is expected to appear as, or increase in, attenuation and volume after intravenous contrast administration, often with progressive accumulation between the arterial and venous phases [1].

By contrast, vicariously excreted material is already present before the new contrast injection and usually shows no meaningful interval increase during the enhanced phases. Associated gallbladder opacification, diffuse rather than focal bowel distribution, persistent nephrograms, and the absence of a compatible clinical manifestation such as hematemesis, melena, or continued hemodynamic deterioration further support VCME. Gallbladder opacification is among the best-recognized manifestations of vicarious excretion [12]. Contrast secreted into bile may accumulate within the gallbladder and subsequently pass through the biliary tract into the small intestine. Depending on the timing of imaging, the findings may, therefore, range from isolated gallbladder hyperattenuation to simultaneous opacification of the gallbladder and bowel. The appearance may persist for hours or occasionally days after the original administration of contrast medium [11]. This temporal behavior explains why the phenomenon is often encountered unexpectedly on follow-up CT rather than on the initial examination.

Persistent nephrograms provide additional context for delayed contrast clearance. Katzberg et al. demonstrated focal or global persistent nephrograms on unenhanced CT 24 hours after cardiac catheterization and found associations with procedural fluoroscopy time and the amount of contrast administered. Although a persistent nephrogram does not invariably indicate clinically significant renal injury, marked bilateral renal enhancement has been linked to altered renal physiology and, in severe cases, contrast-associated acute kidney injury [13]. 

The literature illustrates the broad range of diagnostic pitfalls created by VCME. Kanabolo et al. described colonic contrast after severe renal trauma that initially suggested a nephroenteric fistula; clinical correlation and subsequent imaging confirmed vicarious excretion instead [10]. Thurtle and Petchey reported intraluminal colonic contrast due to vicarious elimination, emphasizing that bowel opacification may occur despite the absence of oral contrast administration [11]. Earlier trauma reports documented gallbladder and bowel opacification after major renal injury and shock [5]. Collectively, these cases show that VCME is not restricted to one organ system or one method of contrast administration and may occur after intravenous, intra-arterial, or combined exposure.

The interventional context adds a further layer of complexity. Active hemorrhage may be intermittent, and angiography may be negative despite convincing CT evidence and a clinically important hematoma. In such circumstances, empirical embolization of the most likely arterial source may be justified on the basis of hematoma distribution and the presumed mechanism of bleeding. Once embolization has been performed, however, every subsequent hyperdense focus should not automatically be attributed to persistent or recurrent hemorrhage. Comparison with the unenhanced acquisition and review of all prior contrast administrations are essential before considering repeat intervention.

In this case, extensive selective and superselective angiographic evaluation did not demonstrate active extravasation, pseudoaneurysm, or arteriovenous fistula. Empirical gastroduodenal artery embolization was performed according to the topographic distribution and presumed source of hemorrhage. On follow-up CT, the absence of new active extravasation, together with pre-existing hyperattenuating material within the gallbladder and bowel on the unenhanced phase, favored VCME. Recognition of this pattern prevented the imaging appearance from being interpreted as proof of recurrent gastrointestinal bleeding.

The principal clinical lesson is that VCME should be considered whenever unexpected gallbladder or bowel opacification is encountered after repeated contrast-enhanced examinations or endovascular procedures. A structured assessment should include confirmation that no oral or enteral contrast was administered, review of the unenhanced CT phase, comparison of attenuation and volume across phases, assessment for a persistent nephrogram, evaluation of renal function and hemodynamic status, and correlation with clinical evidence of hemorrhage. This approach can establish the diagnosis with confidence and avoid unnecessary invasive management.

Although VCME is generally benign and self-limited, its recognition has substantial practical importance. The finding itself usually requires no specific treatment; management should instead be directed toward the underlying illness and any associated impairment of renal or circulatory function. Familiarity with this uncommon excretory pathway allows radiologists to distinguish a physiological consequence of delayed contrast clearance from true active bleeding, thereby reducing repeat contrast exposure, unwarranted angiography, and potentially harmful intervention.

## Conclusions

VCME represents an uncommon but important diagnostic pitfall following administration of large volumes of iodinated contrast medium during CT and endovascular procedures. Recognition of its characteristic imaging appearance may prevent unnecessary repeat angiography, additional embolization procedures, or surgical exploration in patients with suspected recurrent hemorrhage.
